# International﻿ fisheries threaten globally endangered sharks in the Eastern Tropical Pacific Ocean: the case of the Fu Yuan Yu Leng 999 reefer vessel seized within the Galápagos Marine Reserve

**DOI:** 10.1038/s41598-021-94126-3

**Published:** 2021-07-22

**Authors:** Elisa Bonaccorso, Nicté Ordóñez-Garza, Diana A. Pazmiño, Alex Hearn, Diego Páez-Rosas, Sebastián Cruz, Juan Pablo Muñoz-Pérez, Eduardo Espinoza, Jenifer Suárez, Lauren D. Muñoz-Rosado, Andrea Vizuete, Jaime A. Chaves, Maria de Lourde Torres, Walter Bustos, Danny Rueda, Maximilian Hirschfeld, Juan M. Guayasamin

**Affiliations:** 1 Universidad San Francisco de Quito USFQ, Colegio de Ciencias Biológicas y Ambientales, Quito, Ecuador; 2 Laboratorio de Biología Evolutiva, Instituto Biósfera, Universidad San Francisco de Quito, Quito, Ecuador; 3 Universidad San Francisco de Quito (USFQ) and UNC-Chapel Hill Galapagos Science Center (GSC) Av. Alsacio Northia, Isla San Cristóbal, Galápagos, Ecuador; 4 Independent Researcher, Puerto Ayora, Santa Cruz, Galápagos, Ecuador; 5 Faculty of Science and Engineering, University of the Sunshine Coast, Sunshine Coast, QLD Australia; 6 Dirección del Parque Nacional Galápagos, Puerto Ayora, Galápagos, Ecuador; 7 Pontificia Universidad Católica del Ecuador Sede Manabí, Bahía de Caráquez, Manabí Ecuador; 8 Department of Biology, San Francisco State University, 1600 Holloway Ave, San Francisco, CA USA; 9 James Cook University, Townsville, QLD Australia

**Keywords:** Conservation biology, Ichthyology

## Abstract

Shark fishing, driven by the fin trade, is the primary cause of global shark population declines. Here, we present a case study that exemplifies how industrial fisheries are likely depleting shark populations in the Eastern Tropical Pacific Ocean. In August 2017, the vessel Fu Yuan Yu Leng 999, of Chinese flag, was detained while crossing through the Galápagos Marine Reserve without authorization. This vessel contained 7639 sharks, representing one of the largest seizures recorded to date. Based on a sample of 929 individuals (12%), we found 12 shark species: 9 considered as Vulnerable or higher risk by the IUCN and 8 listed in CITES. Four species showed a higher proportion of immature than mature individuals, whereas size-distribution hints that at least some of the fishing ships associated with the operation may have been using purse-seine gear fishing equipment, which, for some species, goes against international conventions. Our data expose the magnitude of the threat that fishing industries and illegal trade represent to sharks in the Eastern Tropical Pacific Ocean.

## Introduction

Amid the current biodiversity crisis, an era of unprecedented species extinction^1^, our ability to document species loss and ecosystem collapse in marine environments has lagged behind compared with terrestrial ecosystems^2^. This situation is particularly worrisome in the case of sharks, a group with a high proportion of top predators central to marine food web dynamics^3^. Some sharks have a high market value and hence are targeted by fisheries for their fins and other products^4^. Given the economic incentives for exploitation and the difficulties involved in controlling this trade, shark fishing has caused substantial population declines^5^. Consequently, at least 74 species of sharks (16% of shark diversity) are threatened worldwide^6^.

In response to global threats, the Convention on International Trade in Endangered Species of Wild Fauna and Flora (CITES) has identified the international shark fin trade as the primary driver of population declines, protecting 46 shark species under this convention. Further, 26 shark species are listed in Annex 1 of the Memorandum of Understanding on the Conservation of Migratory Sharks (www.cms.int/sharks/en/legalinstrument/sharks-mou), the first global instrument for the conservation of migratory species of elasmobranchs. Thus, management to prevent population collapses of some of the most heavily traded and vulnerable shark species has become a global priority (CITES, https://www.citessharks.org). Moreover, sharks benefit from marine protected areas that preserve habitats and populations^7^, and export biomass to non-protected areas, sustaining the yield of nearby fisheries^8, 9^.

Unfortunately, fisheries management regulations and benefits derived from marine protected areas become ineffective when sharks move into international waters^10^ or are undermined when international or local fleets fish protected species within countries’ exclusive economic zones (EEZs)^11, 12^. Here, we present a case study from the Eastern Tropical Pacific Ocean as an example of how the operation of international fleets may hamper national and international efforts to preserve shark populations worldwide.

Our case is staged in the Galápagos Islands, an archipelago world-renowned for its iconic species and marine environments. Established in 1959, the Galápagos National Park (GNP) expanded to the Galápagos Marine Reserve in 1998^13^, a protected area of about 133,000 km^2^. This reserve has afforded over 20 years of protection for endemic and native marine species and holds at least 36 species of sharks (Supplementary Information Table S1).

On August 13th, 2017, an alert was issued to the Navy of Ecuador from the Monitoring Center of the GNP Directorate regarding a sizeable vessel crossing the Galápagos Marine Reserve. The reefer vessel Fu Yuan Yu Leng 999, of Chinese flag, was detained 12 miles north of Punta Pitt, on San Cristóbal Island. The vessel was escorted to Puerto Baquerizo Moreno, the Galápagos Islands’ political and judicial capital, where an inquiry and subsequent trial began.

Here, we combined morphological identification with molecular barcoding to assess the identity of 12% of the carcasses contained in the vessel Fu Yuan Yu Leng 999 and estimated the size distribution of sharks targeted. We argue that the high shark biomass and the high proportion of endangered species transported by the reefer vessel, as well as the recent operation of international fleets around the Ecuadorian Exclusive Economic Zones, reveal a pattern of sustained exploitation of sharks in this region and, more generally, the Eastern Tropical Pacific Ocean. We discuss our results in terms of their implications for the impact of international fishing fleets on the conservation of shark species.

## Results

**Figure 1:**
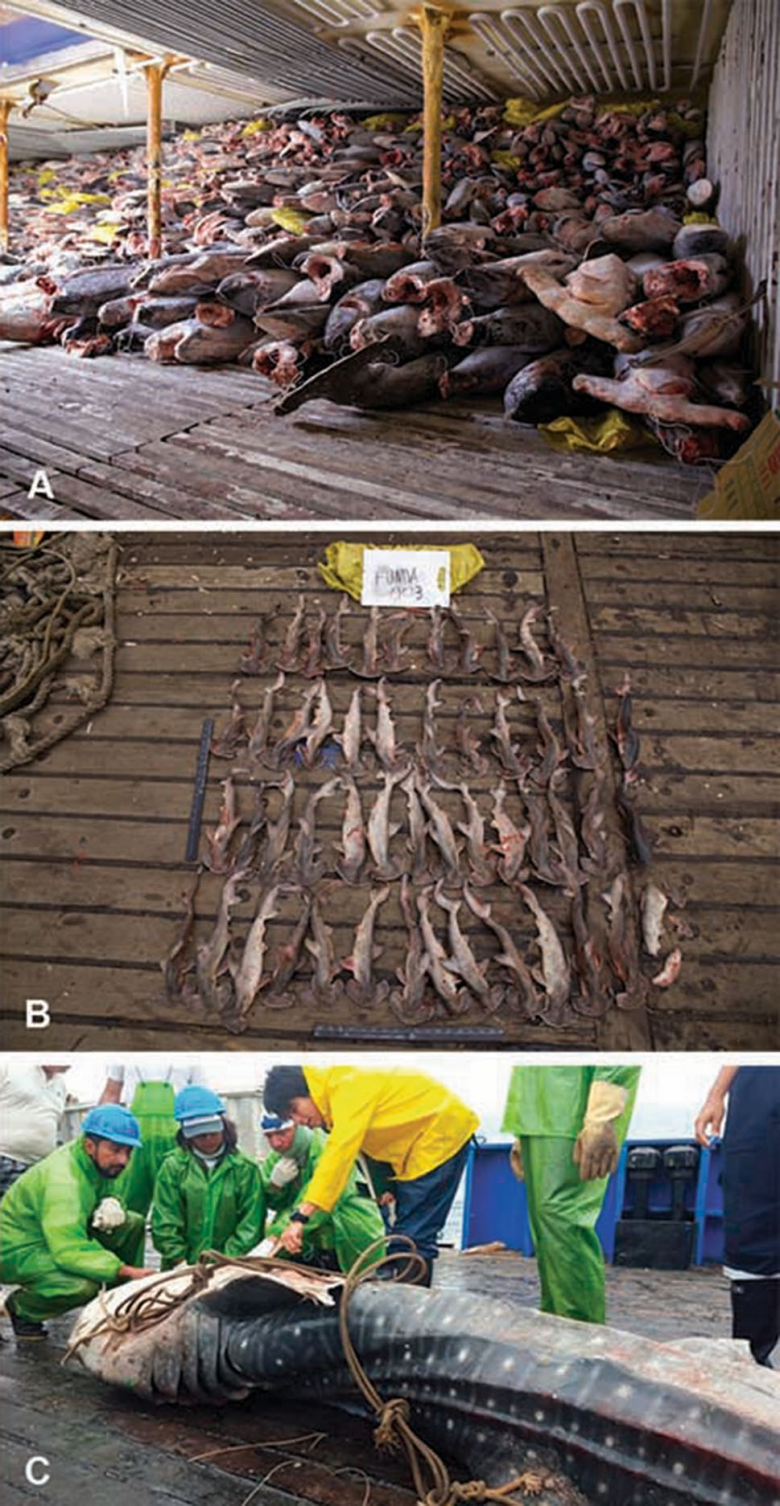
Sample of shark carcasses seized in vessel Fu Yuan Yu Leng 999. A, inside one of the vessel freezers. B, unborn scalloped hammerhead sharks; C, whale shark. We assembled this figure using Adobe Creative Suite (https://www.adobe.com).

The vessel Fu Yuan Yu Leng 999 was carrying 572 tons of fish. The ship contained six freezer holds, and only one and ~ 1/3 were full. The cargo included 7639 sharks (7207 juveniles or adults, 432 unborn; see examples in Fig. 1A,B), 2114 bony fish, and 537 bags of shark fins. Out of the 7639 sharks found, 929 juveniles or adults (12% of the cargo) were morphologically identified and molecularly barcoded (GenBank accession numbers listed in Supplementary Data S1). This sample consisted of 12 species of sharks (Table 1), including a single specimen of the whale shark, *Rhincodon typus* (Fig. 1C).

**Table 1:** Conservation status of species of sharks from vessel Fu Yuan Yu Leng 999 intercepted within the limits of the Galápagos Marine Reserve.

Family/species	Common name	Spanish name	Present in GMR	Total	Frequency	Conservation status IUCN	CITES
**Family Alopidae**							
*Alopias pelagicus*	Pelagic thresher	Tiburón rabón bueno	Yes	122	13.1%	Endangered	Appendix II
*Alopias superciliosus*	Bigeye thresher	Tiburón rabón amargo/ Tiburón zorro ojón	Yes	54	5.8%	Vulnerable	Appendix II
**Family Charcharhinidae**							
*Carcharhinus amblyrhynchos*	Grey reef shark	Tiburón gris de arrecife	Yes	2	0.2%	Endangered	No data
*Carcharhinus falciformis*	Silky shark	Tiburón mico/sedoso	Yes	257	27.7%	Vulnerable	Appendix II
*Carcharhinus longimanus*	Oceanic whitetip shark	Tiburón aletón/ puntas blancas oceánico	Yes	188	20.2%	Critically endangered	Appendix II
*Galeocerdo cuvier*	Tiger shark	Tiburón tigre	Yes	2	0.2%	Near threatened	No data
*Prionace glauca*	Blue shark	Tiburón azul o Aguado	Yes	109	11.7%	Near threatened	No data
**Family Lamnidae**							
*Isurus oxyrinchus*	Shortfin mako	Tiburón tinto	Yes	4	0.4%	Endangered	Appendix II
**Family Rhincodontidae**							
*Rhincodon typus*	Whale shark	Tiburón Ballena	Yes	1	0.1	Endangered	Appendix II
**Family Sphyrnidae**							
*Sphyrna lewini*	Scalloped hammerhead	Cachuda roja	Yes	122	13.1%	Critically endangered	Appendix II
*Sphyrna zygaena*	Smooth hammerhead	Cachuda blanca	Yes	67	7.2%	Vulnerable	Appendix II
**Family Triakidae**							
*Mustelus mustelus*	Common smooth-hound	Tollo	No	1	0.1%	Vulnerable	No data
				929	100%		

Data from www.iucn.org and www.cites.org, updated to December 2020. All species except *Mustelus mustelus* have been reported for the Galápagos Marine Reserve (GMR) (Supplementary Information Table S1).

### Shark identification and geographic affinities

**Figure 2:**
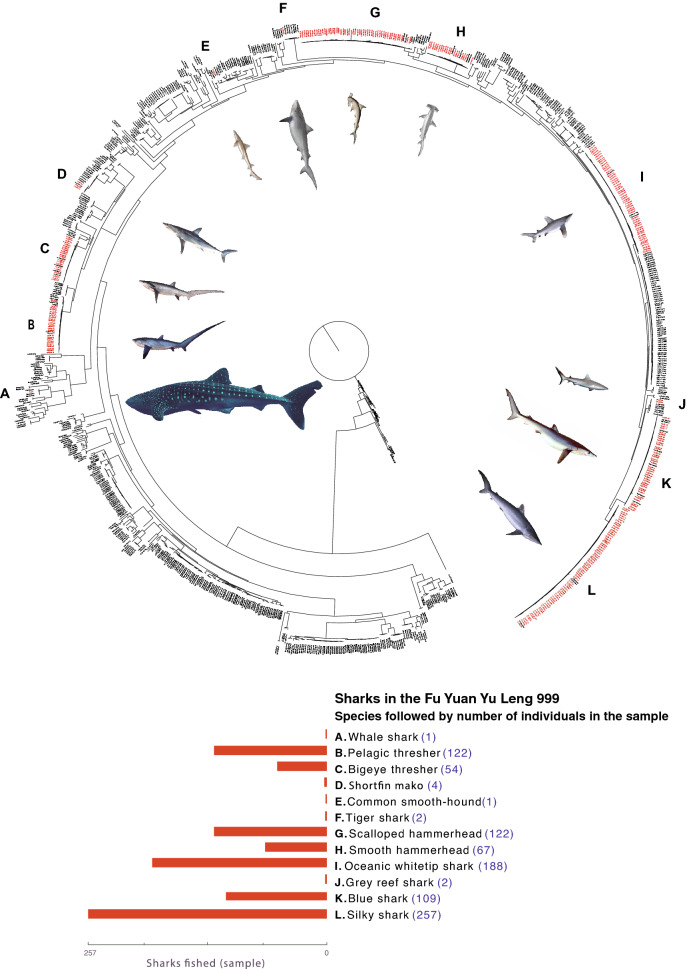
Phylogenetic tree obtained from 929 samples from 12 species of sharks found in vessel Fu Yuan Yu Leng 999, comparative sequences from GenBank (1275 individuals from 229 shark species distributed in the Pacific Ocean), and 9 Galapagos sharks from the Galápagos Islands. The figure also shows the number of identified carcasses per species found in the vessel (a total of 929 individuals); note that these numbers represent only a fraction of the 7639 sharks (7207 juveniles or adults, 432 unborn) found in the Fu Yuan Yu Leng 999. Photo credits: A, Whale shark *(Rhincodon typus)*, Alex Hearn; B, Pelagic thresher *(Alopias pelagicus)*, licenced by CSIRO; C, Bigeye thresher *(Alopias superciliosus)*, licenced by CSIRO; D, Shortfin mako *(Isurus oxyrinchus)*, licenced by CSIRO; E, Common smooth-hound *(Mustelus mustelus)*, Henri Gervais (1877); F, Tiger shark *(Galeocerdo cuvier)*, licenced by CSIRO; G, Scalloped hammerhead *(Sphyrna lewini)*, Henri Gervais (1877); H, Smooth hammerhead *(Sphyrna zygaena)*, Francys Day (1878); I, Oceanic whitetip shark *(Carcharhinus longimanus)*, licenced by CSIRO; J, Grey reef shark *(Carcharhinus amblyrhynchos)*, ReefLifeApps.com, licensed by Creative Commons Attribution-Share Alike 3.0; K, Blue shark *(Prionace glauca)*, licenced by CSIRO; L, Silky shark *(Carcharhinus falciformis)*, CSIRO. We assembled this figure using FigTree 1.4.4 (http://tree.bio.ed.ac.uk/software/figtree/) and Adobe Creative Suite (https://www.adobe.com).

Out of 929 samples analyzed, 800 (86%) matched the morphological and molecular identifications (Supplementary Information Table S2). An overview of the phylogenetic tree is shown in Fig. 2. The complete maximum likelihood tree, with species names and bootstrap supports is available in the Supplementary Information Figure S1, where all samples were located in the same clade as samples of the species assigned by BLAST (bootstrap support ≥ 70%).

We could not determine the geographic affinities between our samples and those retrieved from GenBank because of the limited number of sequences per species from few geographic locations on this database. Also, at least 20% of GenBank sequences were not associated with specific localities and the overall level of individual variation in ND2 was low (see tree in Supplementary Information Figure S1). Still, the 929 sequences generated during this study, 86% of which are backed up by morphological diagnosis, will expand the availability of comparative shark data on GenBank, enhancing species identifications in future studies.

### Conservation status of seized sharks

From the 12 species of sharks found in the vessel Fu Yuan Yu Leng 999, 11 are found within the Galápagos Marine Reserve, 9 are listed as Vulnerable or higher risk by the IUCN (https://www.iucnredlist.org), and 8 are listed as CITES species (Appendix II; http://checklist.cites.org) (Table 1). The species most represented in the cargo were silky shark (Vulnerable; 28%), oceanic whitetip shark (Critically Endangered; 20%), scalloped hammerhead (*Sphyrna lewini*; Critically Endangered; 13%), pelagic thresher (Endangered; 13%), and blue shark (Near Threatened; 12%).

### Size and estimation of size of sexual maturity

**Figure 3:**
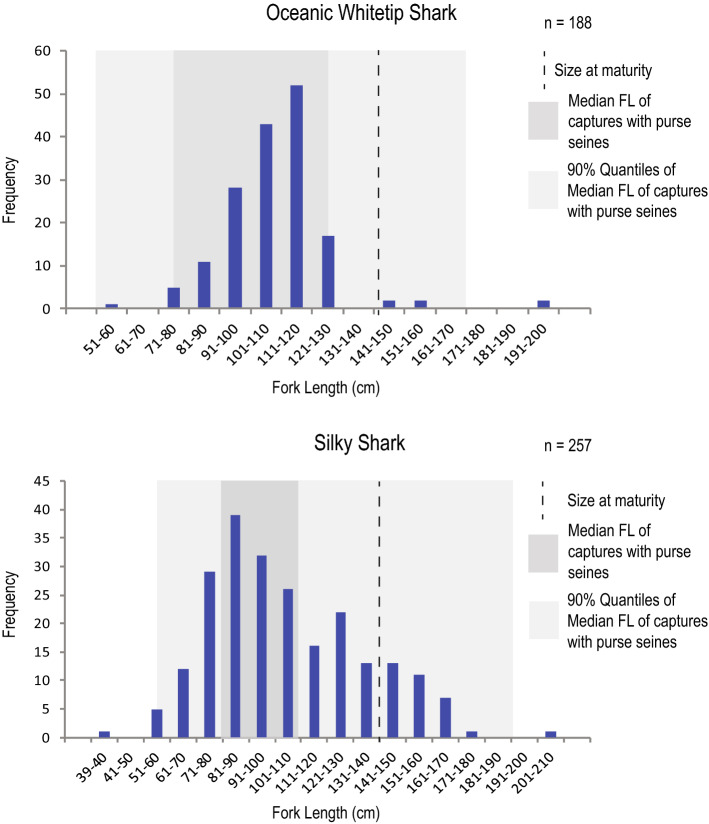
Size structure for oceanic whitetip and silky sharks seized in vessel Fu Yuan Yu Leng 999 (based on 188 and 257 individuals, respectively). Size to maturity for oceanic whitetip was based on Joung et al.^39^, by transforming Total Length to Fork Length^49^. Median Fork Length of captures with purse seines and 90% quantiles were approximated from Clarke et al.^27^ Figure S3. we created this figure using Microsoft Excel Spreadsheet (https://www.microsoft.com/en-ww/microsoft-365/excel) and Adobe Creative Suite (https://www.adobe.com).

We obtained pre-caudal length measurements for 745 individuals of 11 species (Supplementary Data S2). Both sexually mature and immature individuals were present for all species except for tiger shark*,* grey reef shark *Carcharhinus amblyrhynchos*, and common smooth-hound (Table 2). Out of the seven species that had sufficient sampling, four—pelagic thresher, silky shark, oceanic whitetip shark, and blue shark—showed a higher proportion of immature than mature individuals. For oceanic whitetip shark (Critically Endangered), nearly all individuals were immature (96%; 157 out of 164), whereas for silky shark (Vulnerable), immature individuals comprised 86% of the sample (195 out of 228). Size structure for silky and oceanic whitetip sharks is presented in Fig. 3.

**Table 2:** Proportion of sexually mature individuals inferred from pre-caudal length.

Species	Common name	Proportion of immatures	# Sexually immature (SI)	Size range SI (cm)	# Sexually mature (SM)	Size range SM (cm)	Length used	Size of sexual maturity*
*Alopias pelagicus*	Pelagic thresher	55%	52	63.0–137.8	42	139.0–174.0	PCL	138 cm^51^
*Alopias superciliosus*	Bigeye thresher	48%	21	160.2–249.5	23	254.4–302.3	TL	253 cm^52^
*Carcharhinus amblyrhynchos*	Grey reef shark	–	0	NA	2	132.9–143.6	TL	116.7^53^
*Carcharhinus falciformis*	Silky shark	86%	195	37.0–133.0	33	136.0–195.0	PCL	135–140 cm^38^
*Carcharhinus longimanus*	Oceanic whitetip shark	96%	157	76.7–169.9	7	189.1–257.6	TL	172 cm^39^
*Galeocerdo cuvier*	Tiger shark	–	2	145.1–212.3	0	NA	TL	276 cm^54^
*Prionacea glauca*	Blue shark	86%	58	96.8–180.8	9	182.1–199.2	TL	182 cm^55^
*Isurus oxyrinchus*	Shortfin mako	–	2	120.4–171.2	1	203.4	TL	180 cm^56^
*Sphyrna lewini*	Scalloped hammerhead	27%	25	117.0–162.0	69	163.3–295.3	TL	162–181.2 cm^57^
*Sphyrna zygaena*	Smooth hammerhead	7%	3	118.4–172.8	43	179.7–289.9	TL	178.1^58^
*Mustelus mustelus*	Common smooth-hound	–	0	NA	1	161.9	TL	88 cm^59^
*Total*			515		230			

References are for studies reporting size for sexual maturity for males.

*PCL* precaudal length, *TL* total length.

### The legal case against vessel Fu Yuan Yu Leng 999

The provincial court of Galápagos, on San Cristobal Island, ruled in favor of the Galápagos National Park Directorate, imposing legal sanctions on the Fu Yuan Yu Leng 999 vessels’ owner and crew. The process’ legal base rested on the Ecuadorian law prohibiting the unauthorized possession and transport of protected species and on trespassing into the Galápagos Marine Reserve without authorization^14^. The final indictment resulted in the incarceration for 1–3 years of 20 crew members, confiscation of the vessel, and a fine of US$ 6.1 million for damages inflicted to protected species^15^.

## Discussion

The case of vessel Fu Yuan Yu Leng 999 represents the largest reported shark seizure in Ecuadorian waters to date. It reveals an unprecedented magnitude of targeted shark fisheries and highlights the vulnerability of crew members employed by international fishing fleets. Although based on a single case, the fisheries catch reported here exemplifies ongoing industrial operations that are likely depleting shark populations in the region on a vast scale. This situation might have profound implications for the management and conservation of shark populations in the Eastern Tropical Pacific.

**Figure 4:**
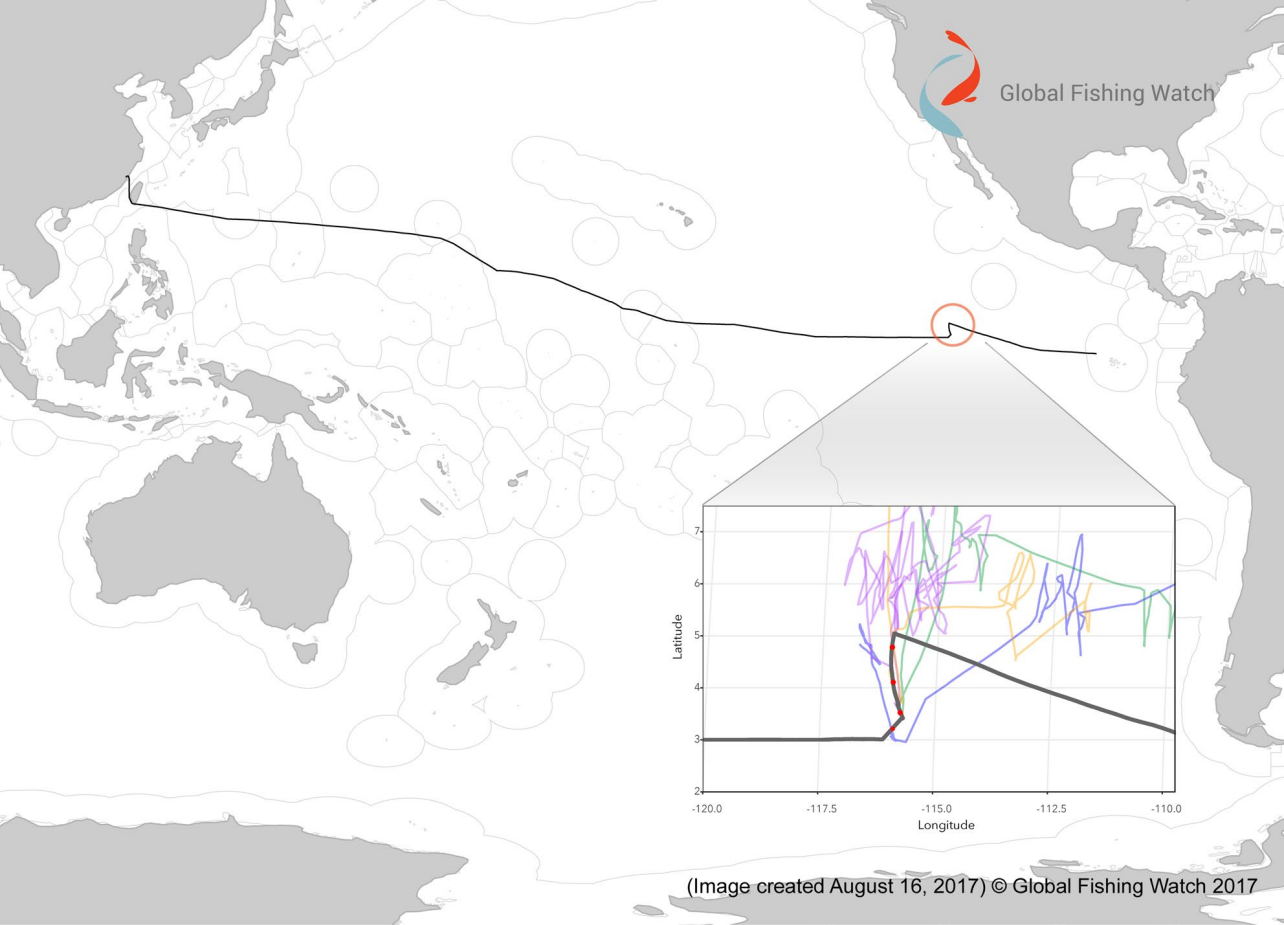
Trajectory of vessel Fu Yuan Yu Leng 999, including AIS–detected anomaly. The vessel departed from Fuzhou on the Chinese coast^50^ and was detained at Punta Pitt, San Cristobal Island, within the Galápagos Marine Reserve. Figure from Cutlip^17^, its use authorized by Global Fishing Watch.

There is no certainty about the origin of sharks found in the vessel Fu Yuan Yu Leng 999. However, machine-learning analysis of the data from the Automatic Identification System (AIS) of the vessel detected an anomaly in its trajectory 2735 km northwest of the Galápagos Marine Reserve^16^ (Fig. 4). Estimated locations of vessel rendezvous coincide with the movements of four Chinese flagged tuna long-liners, suggesting that the Fu Yuan Yu Leng 999 loaded cargo in the area without authorization, making the operation illegal^17^. These inferences are in line with the statements of crew members during legal procedures. Also, it is possible that some of the long-liners trespassed other countries’ economic exclusive zones (EEZ) or received catch from coastal fishing ships. Our finding of two grey reef sharks, a non-pelagic species found in the western Pacific and the Central Pacific Islands^18^, supports this scenario. These detection anomalies highlight the importance of satellite technologies, such as AIS and vessel monitoring systems (VMS), in inferring true vessel trajectories, enforcing approved routes, and diminishing the probability of vessels trespassing into EEZs and marine protected areas^19^.

Whether the Fu Yuan Yu Leng 999 vessel would engage in potentially illegal operations around the Galápagos EEZ, remains unclear. Still, in its empty freezer holdings, the vessel had a gross capacity for at least 4.5 times the number of sharks found in this study (more than 30,000 additional individuals), which may have been received by the vessel along its south-bound trajectory if not been seized.

The number of individuals analyzed in this study constitutes a representative sample of the Fu Yuan Yu Leng 999 vessel’s total cargo, which was highly biased towards sharks. The most common species found in the sample, the silky shark (28%), has been reported as the second most abundant species in the Hong Kong and Guangzhou markets in China, the largest shark fin retail centers in the world^20^. Even more worrisome is the situation of the oceanic whitetip shark (20% of the sample), which is Critically Endangered. In 2011, in response to concerns about declining trends in catches of oceanic whitetip sharks, a ban by the Inter-American Tropical Tuna Commission (IATTC) was placed on the retention, transshipping, landing, storing, or selling of this species (Resolution C-11-10^21^). Thus, our finding of 188 oceanic whitetip sharks implies that the Fu Yuan Yu Leng 999 crew was violating the ban of the IATTC, of which China is a signatory.

Another common species in the sample, the blue shark (12%), has been reported as the most abundant species in Asian markets^20^. This species is not yet been listed by CITES because of its Near Threatened status (i.e., close to qualify for a threatened category in the near future; https://www.iucnredlist.org/). However, regionally, the blue shark represents the most common shark species landed in Peru^22^, and has been reported as by-catch in fisheries from Ecuador^23^ and Chile^24^. Other endangered shark species, such as hammerheads (collectively 20% of the sample), are highly vulnerable to non-target fisheries in the Eastern Pacific Ocean^23, 25, 26^.

Additional threads may derive from the high proportion of immature individuals for some species in the sample. A comparison of fork length distribution of the seized silky and oceanic whitetip sharks (Fig. 3) with size-structured catch data from industrial fleets in the region^27^, suggests that these species may have been caught using purse-seine gear. Consistent with these data, the semi-industrial longline fleet from mainland Ecuador caught mostly adults of silky shark and only a small proportion of oceanic whitetip shark in 2008–2012^23, 28^. Samples of both species of hammerhead shark in the vessel were comprised mostly of adults, which is also consistent with the use of purse-seine gear^29^. In 2016, the IATTC placed a precautionary ban for 2017–2019 on retention, transshipment, landing, or storing of silky sharks caught by purse seines in the convention area (Resolution C-16-06^30^). Thus, our data, which suggest fishing a high proportion of silky sharks with purse seines, points to yet another violation of the IATTC by the Fu Yuan Yu Leng 999 crew. These data exemplify how the operation of international fishing fleets may be hampering the efforts of other IATTC members to protect shark species.

In recent years, international fleets comprising hundreds of vessels have been reported repeatedly near the Ecuadorian EEZs (Galápagos and Continental EEZs). In July 2017 and August 2017 (a few days before the seizure of vessel Fu Yuan Yu Leng 999), international fleets of ~ 300 ships (mostly Chinese flagged) were detected in the international waters bordering the Galápagos’ EEZ^31^ and a similar event happened in March 2019^32^. More recently, on 16 July 2020, a fleet of 260 ships (mostly Chinese flagged) was reported along the southern border of Galápagos’ EEZ^33^. By 6 August 2020, the fleet had grown to 340 ships^34^, and at least 6 were transmitting false coordinates that located them within New Zealand’s waters^35^.

These events suggest that the presence of international fishing fleets around the Ecuadorian EEZs may be systematic. Given the highly mobile nature of the targeted species identified here, international fleets are likely catching sharks that are demographically linked to populations protected under national legislation, undermining the efforts Ecuador has adopted for the conservation of sharks. Current proposals to enhance the protection of migratory marine life in the region include expanding Ecuador’s EEZ along the Carnegie and Cocos ridges (Supplementary Information Figure S2). However, the seasonal return of large industrial fleets to the exact borders of the Ecuadorian EEZs implies that the resources within these zones are of particular interest.

The high seas in the Eastern Tropical Pacific, including those surrounding the Galápagos EEZ, harbor marine resources (in this case, sharks), which are targeted by international fishing fleets, with limited governance from Regional Fisheries Management Organizations (RFMOs). A better understanding of the demographic and genetic connectivity of targeted shark populations across jurisdictional boundaries is urgently needed to inform management and improve the conservation of the biological marine resources of the Eastern Tropical Pacific both within EEZs and in Areas Beyond National Jurisdiction (ABNJs).

Here, we provide the largest data set on shark species caught in the region and contribute to a growing data base of genetic information needed to assess the identity of fished sharks in the future. Improved governance of fisheries in ABNJs and their compliance with international treaties, will be paramount to preserve biological and economical marine resources of developing countries in the Eastern Tropical Pacific and tropical seas in general.

## Methods

### Sampling, morphological identification, and size of sexual maturity

Sampling was conducted onboard vessel Fu Yuan Yu Leng 999 in September and October 2017, in a collaborative effort between the National Army of Ecuador, the GNP Directorate, and the Galapagos Science Center (GSC) of Universidad San Francisco de Quito and the University of North Carolina at Chapel Hill (research permits PC-38-16 and PC-24-17). The procedure was performed under the time and logistic constraints imposed by the seizure’s legal process, the low temperatures of the vessel holds, and the identifying and measuring of frozen samples—with limited personnel and the vessel in motion—while destruction/disposal of the cargo was taking place.

We tried to take a representative sample of all species present in the vessel, choosing a random sample of individuals within each species. Morphological identification was based on expert’s knowledge and specialized literature^18, 28^. Tissue samples and pre-caudal body length were taken for as many individuals as possible. The total vessel cargo was weighed to the nearest 0.1 kg before disposal. The size of sexual maturity was inferred from the literature to estimate the ratio of immature-to-mature individuals in our sample. For pelagic thresher (*Alopias pelagicus*) and silky shark (*Carcharhinus falciformis*), size of sexual maturity was inferred from pre-caudal length. For other species, we used conversion factors to calculate total length from pre-caudal length, as follows: for shortfin mako (*Isurus oxyrinchus*), blue shark (*Prionace glauca*), and smooth hammerhead (*Sphyrna zygaena*) we followed Mas et al.^36^; for oceanic whitetip (*Carcharhinus longimanus*) D´Alberto et al.^37^; for bigeye thresher (*Alopias superciliosus*), grey reef shark (*Carcharhinus amblyrhynchos*), tiger shark (*Galeocerdo cuvier*), and smooth-hound (*Mustelus mustelus*), FishBase (https://www.fishbase.de). For scalloped hammerhead (*Sphyrna lewini*), we used the conversion factor for smooth hammerhead^36^. Since we lacked sex ID for all individuals, we defined minimum size at maturity using estimates for males only (which tend to be smaller than females). When a range was provided, we used the minimum size.

To compare the size structure of silky and oceanic whitetip sharks found in the vessel with that of sharks fished with long-lines and purse-seines^27^, we converted pre-caudal length to fork length using Oshitani et al.^38^ and Joung et al.^39^ conversion factors, respectively.

### Molecular identification

We sequenced the mitochondrial gene Nicotinamide Adenine Dehydrogenase subunit 2 (ND2), a barcode gene for sharks^40^ (permits MAE-DNB-CM-2016-0041 and MAE-DNB-2018-0759-O). We conducted genomic DNA extractions on samples of subcutaneous muscle tissue (2 × 2 mm). Extraction followed a guanidine thiocyanate protein precipitation plus isopropanol DNA precipitation protocol^41^. Quality and concentration of genomic DNA were measured with a NanoDrop ND-1000 spectrophotometer v3.0.1 (NanoDrop, ThermoFisher Scientific) or an Epoch Microplate Spectrophotometer (BioTek).

The ND2 gen was amplified by PCR in 25 μl reactions comprised of 0.25 μl Paltinum Taq DNA Polymerase, Master Mix (Invitrogen), 2.50 μl 10X PCR Buffer, 1.5 mM of MgCl_2_, 0.50 μl of 10 μM of each dNTP, 0.5 μl of each primer (10 μM), and different amounts of ultrapure water with 1 μl of template DNA (adjusted to 100 ng/mL of DNA). Thermocycling conditions included an initial denaturation at 94 °C/3 min; 35 cycles of denaturation at 93 °C/ 30 s, annealing at 58 °C/1 min, and extension at 72 °C/1 min; and a final extension at 72 °C/10 min. The resulting amplicons were visualized via electrophoresis in a 2% agarose gel (UltraPure Agarose). Excess nucleotides and dNTPs were removed from PCR products using ExoSAP-IT PCR Product Cleanup Reagent (Applied Biosystems). Purified amplicons were sequenced with big-dye chemistry and PCR primers, using capillary electrophoresis in an ABI3730xl sequencer. Inspection of chromatograms, contig assembly of forward and reverse sequences, and final sequence checking and edition were performed in Geneious 11.1.5 (Biomatters Ltd.^42^).

To evaluate the similarity between sequences from the vessel’s samples and those on GenBank (https://www.ncbi.nlm.nih.gov/genbank/), we used BLAST (https://blast.ncbi.nlm.nih.gov/Blast.cgi). To place the sample sequences in a phylogenetic framework, we consulted the Chondrichthyes Tree of Life (https://sharkrays.org) to assemble a list including all shark species from the Pacific Ocean, and downloaded the sequences for these species from Genbank. Locality information for sequences of the species present in the vessel was gathered, when possible, in an attempt to relate the seized samples to general geographic regions in the Pacific Ocean. Additionally, we sequenced samples from nine individuals of the Galapagos shark, *Carcharhinus galapagensis*, obtained from the Galápagos Marine Reserve (GNPD permit 065-2013).

Sequences of samples from vessel Fu Yuan Yu Leng 999, the Pacific Ocean (gathered from GenBank), and Galapagos shark were aligned using MAFFT 7^43^. All sequences were inspected, translated into amino acids (to look for potential stop-codons), and trimmed to the size of the ND2 mitochondrial gen using Mesquite 3.6^44^. To reduce the size of our matrix and facilitate phylogenetic analyses, we eliminated duplicates (identical sequences) in all datasets separately, using sRNAtoolbox^45^. Then, we compiled the unique sequences from each dataset and re-aligned them in MAFFT 7.

We used IQ-TREE^46^, as implemented in the IQ-TREE web server^47^ (http://iqtree.cibiv.univie.ac.at/) to infer the best-fit model of nucleotide substitution, estimate the maximum likelihood tree, and assess nodal support. The best-model fit was estimated using ModelFinder^48^ in “Auto” function and “FreeRate heterogeneity”; the best-fit model (GTR + F + R7) was chosen using the Bayesian information criterion. Nodal support was obtained using 1000 ultrafast bootstrap replicates, 1000 maximum iterations, and minimum correlation coefficient of 0.99. As outgroup, we used the rabbit fish, *Chimaera monstrosa* (GenBank accession number JQ518716).

## Supplementary Information

Supplementary Figure S1.

Supplementary Figure S2.

Supplementary Table S1.

Supplementary Table S2.

Supplementary Data S1.

Supplementary Data S2.

## Data Availability

All the DNA sequences generated during this study are available on GenBank (Supplementary Data S1). All morphological data used is available from the Supplementary Information Data S1.
