# Supplementary material for: International﻿ fisheries threaten globally endangered sharks in the Eastern Tropical Pacific Ocean: the case of the Fu Yuan Yu Leng 999 reefer vessel seized within the Galápagos Marine Reserve
Source: Sci Rep. 2021 Jul 22;11:14959. doi: 10.1038/s41598-021-94126-3 (PMC8298506; doi:10.1038/s41598-021-94126-3)
Supplement: Supplementary file 2 — Supplementary Figure S2. [file 41598_2021_94126_MOESM2_ESM.pdf]

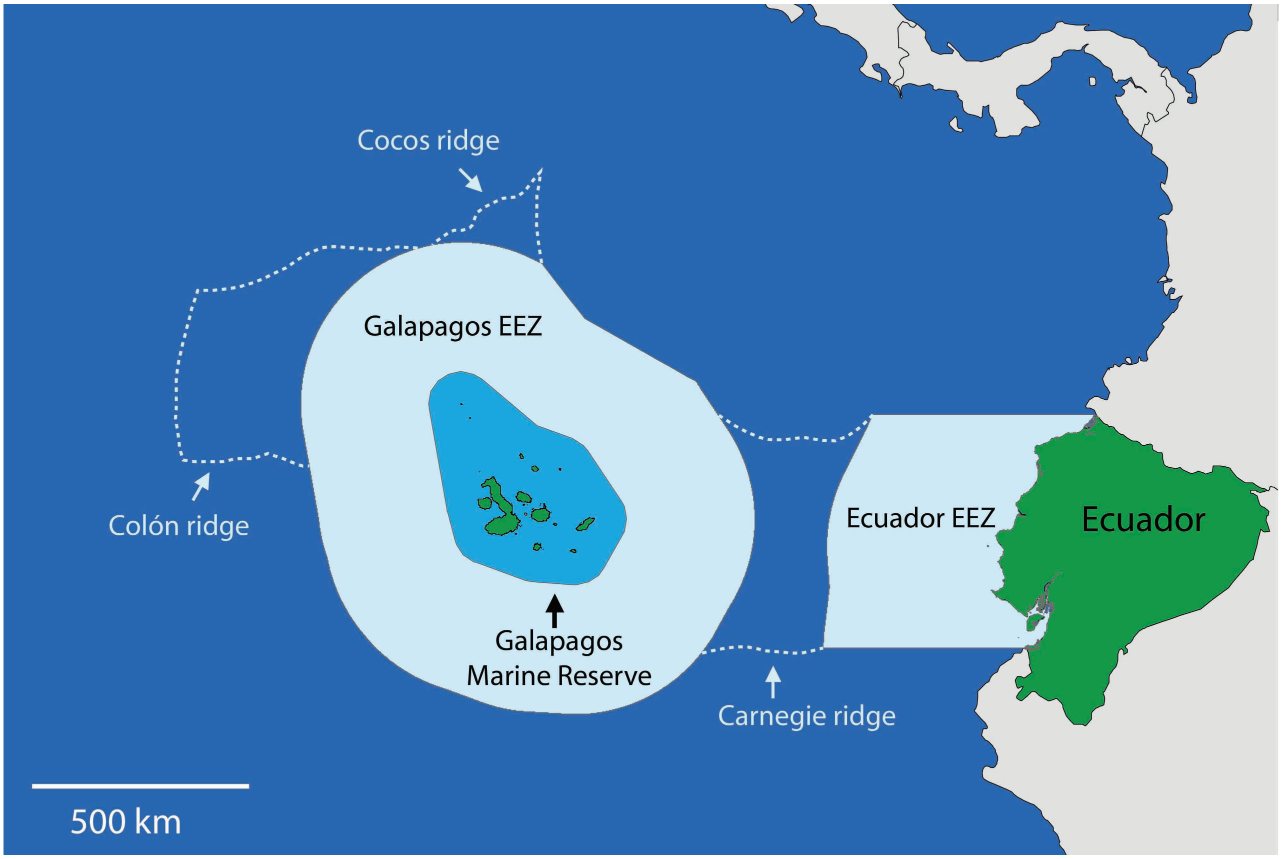

Supplementary Information Figure S2. The Ecuador and Galápagos EEZs (light blue), and proposed extensions of both EZZs (dashed lines).
