# Supplementary material for: International﻿ fisheries threaten globally endangered sharks in the Eastern Tropical Pacific Ocean: the case of the Fu Yuan Yu Leng 999 reefer vessel seized within the Galápagos Marine Reserve
Source: Sci Rep. 2021 Jul 22;11:14959. doi: 10.1038/s41598-021-94126-3 (PMC8298506; doi:10.1038/s41598-021-94126-3)
Supplement: Supplementary file 3 — Supplementary Table S1. [file 41598_2021_94126_MOESM3_ESM.pdf]

Supplementary Information Table S1. Shark species found in the Galapagos Marine Reserve.

Family	Scientific name	Common name	Source	Habitat	Abundance	IUCN status (Dec 2020)	CITES (Jul 2020)
Carcharhinidae	*Carcharhinus galapagensis*	Galapagos shark	Zarate 2002; Hearn et al. 2014; CDF 2019.	coastal	common	Least concern	No data
Carcharhinidae	*Carcharhinus amblyrhynchos*	Grey Reef shark	Zarate 2002.	coastal-pelagic	rare	Endangered	No data
Carcharhinidae	*Carcharhinus limbatus*	Blacktip shark	Zarate 2002; Hearn et al. 2014; CDF 2019.	coastal-pelagic	common	Near threatened	No data
Carcharhinidae	*Carcharhinus albimarginatus*	Silvertip shark	Zarate 2002; Hearn et al. 2014; CDF 2019.	coastal-pelagic	rare	Vulnerable	No data
Carcharhinidae	*Carcharhinus falciformis*	Silky shark	Zarate 2002; Hearn et al. 2014; CDF 2019.	oceanic-epipelagic	common	Vulnerable	Appendix II
Carcharhinidae	*Carcharhinus altimus*	Bignose shark	Zarate 2002; Hearn et al. 2014; CDF 2019..	pelagic	rare	Near Threatened	No data
Carcharhinidae	*Carcharhinus plumbeus*	Sandbar shark	Zarate 2002; Hearn et al. 2014; CDF 2019	coastal-pelagic	rare	Vulnerable	No data
Carcharhinidae	*Carcharhinus longimanus*	Oceanic whitetip shark	Zarate 2002; Hearn et al. 2014; CDF 2019.	oceanic-pelagic (ocasionally coastal)	rare	Critically endangered	Appendix II
Carcharhinidae	*Triaenodon obesus*	Whitetip reef shark	Zarate 2002; Hearn et al. 2014; CDF 2019.	Benthic	common	Vulnerable	No data
Carcharhinidae	*Galeocerdo cuvier*	Tiger shark	Zarate 2002; Hearn et al. 2014; CDF 2019.	coastal-pelagic	common	Near threatened	No data
Carcharhinidae	*Prionace glauca*	Blue shark	Zarate 2002; Hearn et al. 2014; CDF 2019.	coastal-oceanic	common	Near threatened	No data
Carcharhinidae	*Nasolamia velox*	Whitenose shark	Zarate 2002; Hearn et al. 2014; CDF 2019.	coastal-pelagic	rare	Endangered	No data
Sphyrnidae	*Sphyrna zygaena*	Smooth hammerhead	Zarate 2002; Hearn et al. 2014; CDF 2019.	coastal-pelagic	rare	Vulnerable	Appendix II
Sphyrnidae	*Sphyrna lewini*	Scalloped Hammerhead	Zarate 2002; Hearn et al. 2014; CDF 2019.	coastal-pelagic	common	Critically endangered	Appendix II
Sphyrnidae	*Sphyrna tiburo*	Bonnethead shark	Hearn et al. 2014; CDF 2019.	coastal	rare	Critically endangered	No data
Triakidae	*Mustelus mento*	Speckled smoothhound	Zarate 2002; Hearn et al. 2014; CDF 2019.	Benthic		Critically endangered	No data
Triakidae	*Mustelus albipinnis*	White-margin fin houndshark	Acuña-Marrero et al. 2013; Hearn et al. 2014; CDF 2019.	Benthic		Least concern	No data
Triakidae	*Triakis maculata*	Spotted houndshark	Zarate 2002; Hearn et al. 2014; CDF 2019.	Benthic		Critically endangered	No data

Family	Scientific name	Common name	Source	Habitat	Abundance	IUCN status (Dec 2020)	CITES (Jul 2020)
Scyliorhinidae	*Apristurus kampae*	Longnose cat shark	Zarate 2002; Hearn et al. 2014; CDF 2019.	Benthic		Data deficient	No data
Scyliorhinidae	*Apristurus stenseni*	Cat shark	Zarate 2002; Hearn et al. 2014; CDF 2019.	Benthic		Least concern	No data
Scyliorhinidae	*Bythaelurus giddingsi*	Galapagos cat shark	McCosker et al. 2012; Hearn et al. 2014; CDF 2019.	Benthic		Least concern	No data
Scyliorhinidae	*Galeus sp.*	Cat shark	Hearn et al. 2014; CDF 2019.	-		-	-
Lamnidae	*Alopias superciliosus*	Bigeye thresher shark	Zarate 2002; Hearn et al. 2014; CDF 2019.	coastal-oceanic	possibly common	Vulnerable	Appendix II
Lamnidae	*Alopias vulpinus*	Common thresher shark	Zarate 2002.	coastal-oceanic	rare	Vulnerable	Appendix II
Lamnidae	*Alopias pelagicus*	Pelagic thresher shark	Zarate 2002; Hearn et al. 2014; CDF 2019.	oceanic-epipelagic	common	Endangered	Appendix II
Lamnidae	*Isurus paucus*	Longfin mako	Zarate 2002.	oceanic-pelagic	rare	Endangered	Appendix II
Lamnidae	*Isurus oxyrinchus*	Shortfin mako shark	Zarate 2002; Hearn et al 2014.	coastal-oceanic	rare	Endangered	Appendix II
Squalidae	*Centroscyllium nigrum*	Combtooth shark	Zarate 2002; Hearn et al. 2014; CDF 2019.	Benthic		Least concern	No data
Squalidae	*Isistius brasiliensis*	Cookie cutter shark	Zarate 2002; Hearn et al. 2014; CDF 2019.	coastal-oceanic		Least concern	No data
Rhincodontidae	*Rhincodon typus*	Whale shark	Zarate 2002; Hearn et al. 2014; CDF 2019.	coastal-oceanic	common	Endangered	Appendix II
Centrophoridae	*Centrophorus squamosus*	Deepwater spiny dogfish	Acuña-Marrero et al. 2013; Hearn et al. 2014; CDF 2019.	demersal and pelagic	deep	Endangered	No data
Echinorhinidae	*Echinorhinus cookei*	Prickly shark	Hearn et al. 2014; CDF 2019.	Benthic		Data deficient	No data
Odontaspididae	*Odontaspis ferox*	Smalltooth sand tiger shark	Acuña-Marrero et al. 2013; Hearn et al. 2014; CDF 2019.	Benthic	possibly common	Vulnerable	No data
Heterodontidae	*Heterodontus quoyi*	Galapagos bullhead shark	Zarate 2002; Hearn et al. 2014; CDF 2019.	Benthic	common	Least concern	No data
Hexanchidae	*Notorynchus cepedianus*	Broadnose seven-gilled shark	Buglass et al. 2020.	Neritic, oceanic		Vulnerable	No data
Hexanchidae	*Hexanchus griseus*	Bluntnose six-gilled shark	Buglass et al. 2020.	Deep Benthic		Near threatened	No data

References

Acuña-Marrero, D., Zimmerhackel, J.S., Mayorga, J., & Hearn, A. (2013). First record of three shark species, *Odontaspis ferox*, *Mustelus albipinnis* and *Centrophorus squamosus*, from the Galápagos Islands. *Marine Biodiversity Records,* 6 https://doi.org/10.1017/S1755267213000596

Buglass, S., Nagy, S., Ebert, D., Sepa, P., Turchik, A., Bell, K. L., Rivera, F., & Giddens J. (2020). First records of the seven-gilled *Notorynchus cepedianus* and six-gilled *Hexanchus griseus* sharks (Chondrichthyes: Hexanchiformes: Hexanchidae) found in the Galápagos Marine Reserve. *Journal of Fish Biology,* 97, 926–929.

Fundación Charles Darwin. (2009). Tiburones de la Reserva Marina de Galápagos. Puerto Ayora, Islas Galápagos, Ecuador: Fundación Charles Darwin.

Hearn, A. R., Acuña, D., Ketchum, J. T., Penaherrera, C., Green, J., Marshall, A., ... Shillinger, G. (2014). Elasmobranchs of the Galapagos Marine Reserve: A Dynamic Social-Ecological System. In J. Denkinger & L. Vinueza (Eds.), The Galapagos marine reserve (pp. 23–59) Springer, Cham. https://doi.org/10.1007/978-3-319-02769-2_2

McCosker, J. E., Long, D. J., & Baldwin, C. C. (2012). Description of a new species of deepwater catshark, *Bythaelurus giddingsi* sp. nov., from the Galápagos Islands (Chondrichthyes: Carcharhiniformes: Scyliorhinidae). *Zootaxa,* 3221, 48–59.

Zarate, P. (2002). Tiburones. In E. Danulat & G. J. Edgar (Eds.), Reserva Marina de Galápagos: Línea Base de la Biodiversidad (pp. 373–388) Santa Cruz, Ecuador: Fundación Charles Darwin and Servicio Parque Nacional Galápagos.
