# Supplementary material for: International﻿ fisheries threaten globally endangered sharks in the Eastern Tropical Pacific Ocean: the case of the Fu Yuan Yu Leng 999 reefer vessel seized within the Galápagos Marine Reserve
Source: Sci Rep. 2021 Jul 22;11:14959. doi: 10.1038/s41598-021-94126-3 (PMC8298506; doi:10.1038/s41598-021-94126-3)
Supplement: Supplementary file 4 — Supplementary Table S2. [file 41598_2021_94126_MOESM4_ESM.pdf]

Supplementary Information Table S2. Number of shark species from vessel Fu Yuan Yu Leng 999 intercepted within the limits of the Galapagos Marine Reserve, identified with morphology vs. genetic identification using ND2 mitochondrial gene. In bold are species not detected by morphology but identified by ND2 (family indicated if different from that originally assigned). Molecular identifications confirmed by morphology were noted on GenBank by adding this source modifier "Note: molecular identification confirmed by morphological identification".

Morphological ID (n)	Molecular ID		
	**Same as morphological ID**	**Different from morphological ID**	**Different species detected or species ID**
**Family Alopidae**			
Alopias pelagicus (118)	109	9	*A. superciliosus* (4), *C. falciformis* (3), *I. oxyrinchus* (1), *S. lewini* (1)
Alopias superciliosus (51)	46	5	*A. pelagicus* (4), *P. glauca* (1)
**Family Charcharhinidae**			
Carcharhinus albimarginatus (1)	0	1	*C. longimanus* (1)
Carcharhinus falciformis (264)	248	16	*A. pelagicus* (6), *A. superciliosus* (4), *C. longimanus* (2), ***Mustelus mustelus* (1)**, *P. glauca* (1), *S. lewini* (2)
Carcharhinus galapagensis (57)	0	57	***C. amblyrhynchos* (1),** *C. longimanus* (54),<br>***Galeocerdo cuvier* (2)**
Carcharhinus leucas (1)	0	1	*C. longimanus* (1)
Carcharhinus longimanus (116)	108	8	*A. pelagicus* (1), ***C. amblyrhynchus* (1)**, *C. falciformis* (4), *P. glauca* (1), *S. zygaena* (1)
Carcharhinus sp. (24)	22	2	*C. longimanus* (22), *P. glauca* (2)
Prionace glauca (107)	104	3	*C. falciformis* (1), *A.pelagicus* (2)
**Family Lamnidae**			
Isurus oxyrinchus (1)	1	0	
Isurus sp. (1)	1	0	*Isurus oxyrinchus* (1)
**Family Rhincodontidae**			
Rhincodon typus (1)	1	0	
**Family Sphyrnidae**			
Sphyrna lewini (140)	117	23	*S. zygaena* (23)
Sphyrna zygaena (46)	43	3	*C. falciformis* (1), *S. lewini* (2)
Not identified (1)	0	1	*Isurus oxyrinchus* (1)
**Total (929)**	**800**	**129**	
