# Supplementary material for: International﻿ fisheries threaten globally endangered sharks in the Eastern Tropical Pacific Ocean: the case of the Fu Yuan Yu Leng 999 reefer vessel seized within the Galápagos Marine Reserve
Source: Sci Rep. 2021 Jul 22;11:14959. doi: 10.1038/s41598-021-94126-3 (PMC8298506; doi:10.1038/s41598-021-94126-3)

Supplementary Information Figure S1. Maximum Likelihood Phylogenetic tree showing unique sequences of the individuals found in vessel Fu Yuan Yu Leng 999 (in blue), and comparative material from GenBank and Carcharhinus galapagensis from the Galapagos Marine Reserve. Numbers beside codes of samples from the vessel indicate number of individuals found with same sequence.

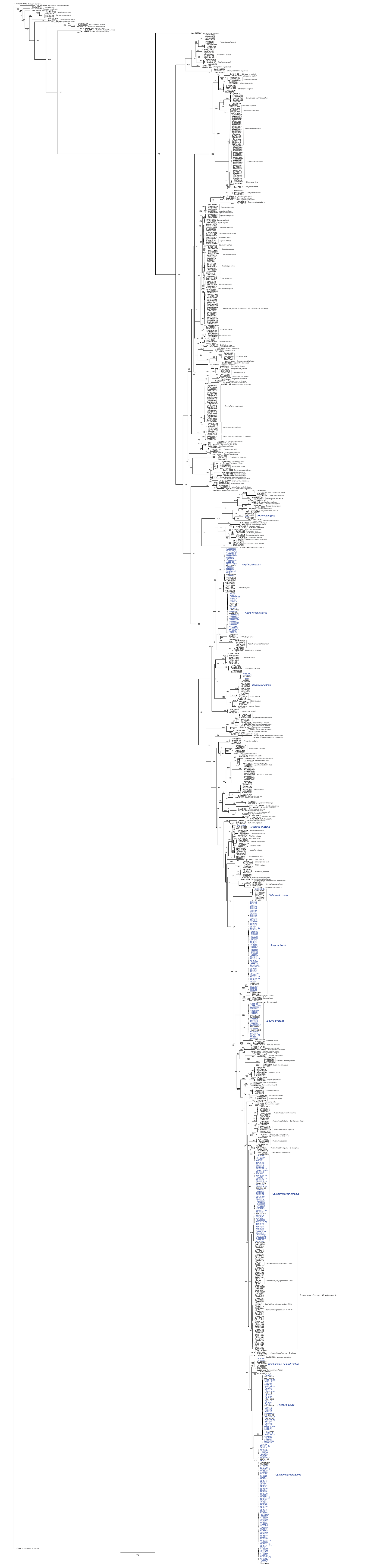

Supplement: Supplementary file 1 — Supplementary Figure S1. [file 41598_2021_94126_MOESM1_ESM.pdf]
